# Chemical Constituents and an Antineuroinflammatory Lignan, Savinin from the Roots of* Acanthopanax henryi*

**DOI:** 10.1155/2019/1856294

**Published:** 2019-02-21

**Authors:** Xiao-Jun Li, Kwan-Woo Kim, Hyuncheol Oh, Xiang-Qian Liu, Youn-Chul Kim

**Affiliations:** ^1^Institute of Pharmaceutical Research and Development, College of Pharmacy, Wonkwang University, Iksan 54538, Republic of Korea; ^2^Hanbang Cardio-Renal Syndrome Research Center, Wonkwang University, Iksan 54538, Republic of Korea; ^3^School of pharmacy, Hunan University of Chinses Medicine, Changsha 4102098, Hunan, China

## Abstract

The phytochemical investigation on the roots of* Acanthopanax henryi* (Araliaceae) resulted in the discovery of twenty compounds whose chemical structures were elucidated by the analysis of 1D-, 2D-NMR, mass spectrometry data, other physicochemical properties, and a comparison of the spectral data with the literature. They were identified as (-)-sesamin (**1**), helioxanthin (**2**), savinin (**3**), taiwanin C (**4**), 6-methoxy-7-hydroxycoumarin (**5**), behenic acid (**6**), 3-*O*-caffeoyl-quinic acid (**7**), 5-*O*-caffeoyl-quinic acid (**8**), 1,3-di-*O*-caffeoyl-quinic acid (**9**), 1,4-di-*O*-caffeoyl-quinic acid (**10**), 1,5-di-*O*-caffeoyl-quinic acid (**11**), (+)-*threo*-(7*R*,8*R*)-guaiacylglycerol-*β*-coniferyl aldehyde ether (**12**), (+)-*erythro*-(7*S*,8*R*)-guaiacylglycerol-*β*-coniferyl aldehyde ether (**13**), ferulic acid (**14**), caffeic acid (**15**), stigmasterol (**16**), *β*-sitosterol (**17**), adenosine (**18**), syringin (**19**), and* trans*-coniferin (**20**). Among these isolates, compound** 3** showed inhibitory activity against lipopolysaccharide- (LPS-) induced nitric oxide (NO) and prostaglandin E2 (PGE_2_) production with IC_50_ values of 2.22 ± 0.11 and 2.28 ± 0.23 *μ*M, respectively. The effects of compound** 3** were associated with the suppression of LPS-induced expression of the inducible nitric oxide synthase (iNOS) and cyclooxygenase-2 (COX-2) protein. Furthermore, compound** 3** negatively regulated the production of interleukin- (IL-) 1*β* and tumor-necrosis factor- (TNF-) *α* at the transcriptional level in LPS-stimulated BV2 microglial cells. These antineuroinflammatory effects of compound** 3** were mediated by p38 mitogen-activated protein kinase (MAPK).

## 1. Introduction

Neuroinflammatory responses are mainly mediated by microglial activation, and they are implicated in the development of neurodegenerative diseases such as Alzheimer's diseases (AD), Parkinson's disease (PD), multiple sclerosis (MS), and amyotrophic lateral sclerosis (ALS) [1]. Microglial cells are the primary resident immune cells in the central nervous system (CNS) and are associated with defensive mechanisms to maintain homeostasis against brain injury [2]. In steady state, microglia exert protective responses by regulating innate and adaptive immune responses. However, activated microglia produce excess immune reactions which are detrimental to brain tissue and can produce various proinflammatory mediators such as tumor necrosis factor- (TNF-) *α*, interleukins (ILs), nitric oxide (NO), prostaglandin E2 (PGE_2_), and reactive oxygen species (ROS). Released and accumulated, these proinflammatory mediators facilitate the development of neurodegenerative diseases [3, 4]. Therefore, it is important to suppress the secretion of proinflammatory mediators from activated microglial cells to prevent the neuroinflammation-related development of neurodegenerative diseases.

*Acanthopanax *spp. is one of the well-known medicinal resources in traditional oriental medicine in China, Korea, Japan, and far-east Russia. Its dried roots and stem barks are famous traditional folk medicine for treating rheumatism, arthritis, paralysis, sinew, and bone pain [5].* Acanthopanax henryi *(Oliv.) Harms, a Chinese endemic plant, has been used as a traditional remedy for the treatment of paralysis, arthritis, rheumatism, lameness, edema, injury from falls, hernia, and abdominal pain [6, 7]. Previous phytochemical studies on* A. henryi* led to the isolation and identification of more than 30 secondary metabolites, including five flavonoids, six caffeoylquinic acid derivatives [6], sixteen triterpenoid saponins [8, 9], one amide, one anthraquinone, one organic acid [9], three lignans, one diterpene, one phenylpropanoid, and two phytosterols [10]. In addition, it has been reported that this plant exhibits diverse pharmacological activities due to the wide variety of chemical constituents. For example, metabolites of the leaves of* A. henryi* have strong antioxidant and antiacetyl cholinesterase activities [6], and the 80% methanol fraction of root bark and ciwujianoside C3, which was isolated from leaves of this plant, have significant anti-inflammatory effect in lipopolysaccharide- (LPS-) induced RAW264.7 macrophage cells [5, 11]. Moreover, some glycosides from the leaves of* A. henryi* have antiadipogenic effect, decreasing lipid accumulation through the inhibition of proliferator-activated receptor gamma (PPAR*γ*) and CCAAT/enhancer-binding protein alpha (C/EBP*α*) in 3T3-L1 cells [12]. However, it has not been investigated yet whether this plant has antineuroinflammatory effects.

Therefore, in this study, we purified twenty compounds from the roots of* A. henryi* and evaluated their antineuroinflammatory activity* in vitro* to continue our approach to contribute to the drug development for inflammation-mediated neurodegenerative diseases.

## 2. Materials and Methods

### 2.1. General Experimental Procedures

NMR spectra (1D and 2D) were recorded using a JEOL JNM ECP-400 spectrometer (Tokyo, Japan) (400 MHz for ^1^H and 100 MHz for ^13^C). HMQC and HMBC experiments were optimized for ^1^*J*_CH_ = 140 Hz and ^n^*J*_CH_ = 8 Hz, respectively. Optical rotations were recorded using a Jasco p-2000 digital polarimeter (Tokyo, Japan). HPLC (YOUNGLIN-YL9100, Younglin, Anyang, Korea) separation was performed on YMC-Pack ODS-A column (20 × 150 mm, 5 *μ*m, and 12 nm) with a flow rate of 5 mL/min and the solvents used for HPLC were analytical grade. ESI-MS data were obtained using a Q-TOF micro-LC-MS/MS instrument (Waters, Milford, MA, USA) at Korea University, Seoul, Korea. TLC was performed on Kieselgel 60 F_254_ (1.05715; Merck, Darmstadt, Germany) or RP-18 F_254s_ (Merck) plates. Spots were visualized by spraying with 10% aqueous H_2_SO_4_ solution followed by heating. Column chromatography (CC) was performed on silica gel (Kieselgel 60, 70-230 mesh and 230-400 mesh, Merck) and YMC octadecyl-functionalized silica gel (C_18_). These procedures were based on our previous report [13].

### 2.2. Plant Materials

The roots of* A. henryi *were collected from its natural habitat in Xinhua, Hunan Province, China, in October 2015 and identified by Professor Xiang Qian Liu, one of the coauthors of this study. A voucher specimen has been deposited in Herbarium of Hunan University of Chinese Medicine, Hunan, China (No. 20151025)

### 2.3. Extraction and Isolation

The air dried, powdered roots of* A. henryi* (10 kg) were extracted with methanol under reflux (3 × 30 L). The solvent was removed under reduced pressure to give a residue (303 g), which was suspended in distilled water and successively partitioned with petroleum ether (PE, b.p. 60-90°C), ethyl acetate (EtOAc), and n-butanol (BuOH), respectively.

The EtOAc extract (41 g) was subjected to CC (7.0 × 60 cm) on silica gel, eluted with a gradient of dichloromethane (CH_2_Cl_2_)-methanol (MeOH) (100:1 to 10:1, v/v) to give ten fractions (Fr. E1-Fr. E10). Fr. E3 (2.630 g) was purified on silica gel CC (2.2 × 70 cm), eluted with hexane-EtOAc (30:1 to 1:1, v/v) to afford** 1** (61.0 mg),** 2** (28.0 mg),** 3** (23.0 mg),** 4** (7.0 mg), and** 5** (5.5 mg). Fr. E5 (444 mg) was firstly subjected to a C_18_ CC (1.2 × 60 cm), with a gradient MeOH-H_2_O (3:7 to 4:6, v/v) as the solvent and then purified by C_18_-Prep-HPLC (CH_3_CN-H_2_O = 1:4, v/v) to achieve** 12** (1.1 mg, t_R_ 25.8 min) and** 13** (1.1 mg, t_R_ 27.5 min). Fr. E7 (668 mg) was firstly subjected to a C_18_ CC (1.8 × 40 cm) elution with MeOH-H_2_O (2:8 to 3:7, v/v) and then further purified by silica gel CC (1.2 × 60 cm) using chloroform (CHCl_3_)-MeOH (20:1 to 10:1, v/v) to obtain colorless needles** 14** (9.0 mg). Fr. E10 (10 g) was subjected to C_18_ CC (4.0 × 50 cm) elution with MeOH-H_2_O (2:8 to 8:2, v/v) to give eight subfractions (Fr. E10.1-Fr. E10.8). Yellow amorphous powder** 7** (82.0 mg) was isolated from Fr. E10.1 (3.207 g) by C_18_ CC (2.2 × 70 cm, MeOH-H_2_O = 1:9, v/v) and decolorization with MeOH. Similarly, yellow amorphous powder** 10** (48.0 mg) was obtained from Fr. E10.3 (1.0 g) by C_18_ CC (1.8 × 80 cm, MeOH-H_2_O = 2:8, v/v) and decolorized with methanol. Compound** 9 **(60.0 mg) was achieved from Fr. E10.5 (1.5 g) by decolorization with methanol.

The PE extract (80 g) was subjected to CC (6.5 × 40 cm) on a silica gel, eluted with a gradient of hexane-EtOAc (10:0 to 1:3, v/v) to give thirteen fractions (Fr. P1-Fr. P13). Fr. P4 (9.920 g) was subjected to a silica gel CC (4.0 × 50 cm) with a hexane-EtOAc gradient (250:1 to 40:1, v/v) as the solvent to gain** 6 **(200.0 mg). Fr. P8 (3.190 g) was firstly performed on a silica gel CC (2.2 × 70 cm) and then purified by recrystallization with hexane-EtOAc (7:1) to afford** 16** (50.0 mg) and** 17** (100.0 mg).

The n-BuOH extract (100 g) was subjected to a C_18_ CC (7.0 × 60 cm) elution with a gradient of MeOH-H_2_O (5:95 to 60:40, v/v) to give nine fractions (Fr. B1-Fr. B9).** 15 **(67.0 mg) was achieved from Fr. B2 by decolorization with MeOH. Fr. B5 (7 g) was firstly fractionated by a C_18_ CC (4.0 × 50 cm) elution with MeOH-H_2_O (12:88 to 40:60, v/v) to afford seven subfractions (Fr. B5.1-B5.7), of which Fr. B5.3 (3 g) was then subjected to silica gel CC (2.2 × 70 cm) to yield** 8 **(71 mg) and** 19 **(26 mg). Fr. B4 (1.5 g) was subjected to silica gel CC (1.8 × 80 cm), eluted with CHCl_3_-MeOH (10:1 to 5:1, v/v) and then purified by successive recrystallizations with MeOH to give** 20** (12 mg). Fr. B3 (2.5 g) was subjected to a silica gel column (2.2 × 70 cm), eluted with a gradient of CHCl_3_-MeOH (10:1 to 8:1, v/v) to obtain six subfractions (Fr. B3.1-B3.6). Colorless crystal** 18** (18 mg) was yielded from Fr. B3.3 (278 mg) by recrystallization with MeOH. Compound** 11 **(4.0 mg) was obtained from Fr. B3.5 (300 mg) by repeated silica gel CC (1.2 × 60 cm) using CHCl_3_-MeOH-H_2_O (3:1:0.1 to 2:1:0.1, v/v/v) as a mobile phase and decolorization with MeOH.

### 2.4. Chemicals and Reagents

Dulbecco's modified Eagle's medium (DMEM), fetal bovine serum (FBS), and other tissue culture reagents were purchased from Gibco BRL Co. (Grand Island, NY, USA). Lipopolysaccharide was obtained from Sigma-Aldrich (St. Louis, MO). Primary antibodies such as anti-inducible nitric oxide synthase (iNOS) and anti-cyclooxygenase-2 (COX-2) antibodies were purchased from Santa Cruz Biotechnology (Dallas, TX, USA). Anti-p-extracellular signal-regulated kinase (ERK), anti-ERK, anti-p-c-Jun N-terminal kinase (JNK), anti-JNK, anti-p-p38, and anti-p38 were obtained from Cell Signaling Technology (Danvers, MA, USA). Anti-mouse, anti-goat, and anti-rabbit secondary antibodies were supplied by Merck Millipore (Darmstadt, Germany). The enzyme-linked immunosorbent assay (ELISA) kit for PGE_2_ was purchased from R&D Systems, Inc. (Minneapolis, MN, USA).

### 2.5. Cell Culture

BV2 microglial cells were received from Professor Hyun Park at Wonkwang University (Iksan, Korea). BV2 cells were maintained at 5 × 10^6^ cells in a 100 mm dish with DMEM supplemented with 10% (v/v) heat-inactivated FBS, penicillin G (100 units/mL), streptomycin (100 *μ*g/mL), and l-glutamine (2 mM) and incubated at 37°C in a humidified atmosphere containing 5% CO_2_.

### 2.6. Determination of Nitrite (NO Production)

BV2 cells were cultured in 24-well culture plates at a density of 5 × 10^4^ cells/well. BV2 cells were pretreated with the isolated compounds from* A. henryi* and then stimulated with LPS (1 *μ*g/mL) for 24 h. After incubation, 100 *μ*L of each supernatant was collected and mixed with the same volume of the Griess reagent. The absorbance at 540 nm wavelength was measured using a microplate reader. The detailed procedures are described in our previous report [14].

### 2.7. PGE_2_ Assay

The level of PGE_2_ present in each sample was determined using a commercially available kit from R&D Systems (Minneapolis, MN, USA). Three independent assays were performed according to the manufacturer's instructions. Briefly, BV2 cells were seeded in 24-well culture plates at a density of 5 × 10^4^ cells/well. Prior to the stimulation with LPS (1 *μ*g/mL) for 24 h, cells were treated with test compounds. After incubation, supernatant was collected and applied to the PGE_2_ ELISA kit for measuring the concentration of PGE_2_.

### 2.8. Western Blotting Analysis

The proteins iNOS, COX-2, and MAPK-associated proteins, including p-p38, p38, p-JNK, JNK, p-ERK, and ERK, were detected by a Western blot analysis. The procedures of this experiment were based on our previous report [14]. Cells were lysed by RIPA buffer (Thermo Fisher Scientific, USA), and normalized for equal amounts of protein using the Bradford protein assay (Bio-Rad Laboratories, Hercules, CA, USA). 30 *μ*g of protein was loaded for each sample and separated on 7.5 or 12% sodium dodecyl sulfate–polyacrylamide gel electrophoresis (SDS-PAGE) gels. Then, proteins were transferred to nitrocellulose (NC) membranes (Bio-Rad Laboratories). After that, membranes were incubated with Tris-buffered saline containing 0.1% Tween-20 (TBS-T) with 5% skim milk (BD Difco, USA) for 1 h at 4°C. Then, membranes were probed with primary antibodies and incubated at 4°C for 90 min or overnight. After incubation, the membranes were washed with TBS-T, and then secondary antibodies were used for detecting primary antibodies. The protein bands on the NC membranes were covered by chemiluminescence with Amersham ECL Prime Western Blotting Detection Reagent (GE Healthcare, Little Chalfont, UK).

### 2.9. Assays for IL-1*β*, IL-6, and TNF-*α*

The culture media were collected to determine the levels of IL-1*β*, IL-6, and TNF-*α* present in each sample using appropriate ELISA kits (R&D Systems, Inc.), as per the manufacturer's instructions. Briefly, BV2 cells were seeded in 24-well culture plates at a density of 5 × 10^4^ cells/well. After incubation, the supernatant was collected and applied to the cytokine ELISA kits for measuring the concentrations of IL-1*β*, IL-6, and TNF-*α*.

### 2.10. Quantitative Real-Time Polymerase Chain Reaction (qRT-PCR)

The detailed procedures of qRT-PCR and the primer sequences, which were used in this investigation were reported in our previous investigations [14, 15]. The analysis was conducted three times independently.

### 2.11. Statistical Analysis

Data are presented as the mean ± standard deviation (SD) of at least three independent experiments. One-way analysis of variance (ANOVA), followed by Tukey's multiple comparison test, was used to compare three or more groups. Statistical analysis was performed using GraphPad Prism software, version 3.03 (GraphPad Software Inc., San Diego, CA, USA).

## 3. Results

### 3.1. Structure Determination

#### 3.1.1. (-)-Sesamin (**1**)

Colorless needles (MeOH), [*α*]_D_^20^ -46.7 (*c* 6.10, CHCl_3_), ESI-MS (*m/z*) 377 [M + Na]^+^; ^1^H-NMR (CDCl_3_, 400 MHz) *δ*: 3.05 (2H, m, H-8, H-8′), 3.86 (2H, dd,* J* = 9.2, 3.6 Hz, H-9a, H-9′a), 4.23 (2H, dd,* J* = 9.2, 6.8 Hz, H-9e, H-9′e), 4.71 (2H, d,* J* = 4.4 Hz, H-7, H-7′), 5.94 (4H, s, 2OCH_2_O), 6.76-6.81 (4H, m, H-5, H-6, H-5′, H-6′), 6.84 (2H, d,* J* = 1.6 Hz, H-2, H-2′); ^13^C-NMR (CDCl_3_, 100 MHz) *δ*:148.1 (C-3, C-3′), 147.2 (C-4, C-4′), 135.2 (C-l, C-1′), 119.4 (C-6, C-6′), 108.3 (C-5, C-5′), 106.6 (C-2, C-2′), 101.2 (2OCH_2_O), 85.9 (C-7, C-7′), 71.8 (C-9, C-9′), 54.4 (C-8, C-8′). Based on a comparison of these ^1^H- and ^13^C-NMR data with data previously reported [16], compound** 1** was identified as (-)-sesamin.

#### 3.1.2. Helioxanthin (**2**)

Light yellow powder, ESI-MS (*m/z*) 371 [M + Na]^+^; ^1^H-NMR (DMSO-*d*_*6*_, 400 MHz) *δ*: 8.55 (1H, s, H-7), 7.94 (1H, d,* J* = 8.8 Hz, H-6), 6.90 (1H, dd,* J* = 1.6, 8.0 Hz, H-6′), 7.51 (1H, d,* J* = 8.8 Hz, H-5), 7.01 (1H, d,* J* = 1.6 Hz, H-2′), 6.96 (1H, d,* J* = 8.0 Hz, H-5′), 6.01, 5.99 (each 1H, d,* J* = 1.2 Hz, H-10), 6.12, 6.07 (each 1H, d,* J* = 0.8 Hz, H-10′), 5.28 (2H, s, H-9′); ^13^C-NMR (DMSO-*d*_*6*_, 100 MHz) *δ*: 171.0 (C-9, C=O), 147.4 (C-4′), 147.3 (C-3′), 147.1 (C-3), 141.7 (C-4), 140.6 (C-8), 130.9 (C-1), 130.6 (C-1′), 128.9 (C-7′), 127.5 (C-7), 126.3 (C-6), 123.3 (C-6′), 121.3 (C-2), 121.2 (C-8′), 112.3 (C-5), 110.4 (C-2′), 108.3 (C-5′), 101.9 (C-10, -OCH_2_O-), 101.7 (C-10′, -OCH_2_O-), 69.9 (C-9′, lactone, -CH_2_-). The data of ^1^H- and ^13^C-NMR were consistent with those in previous report [16]. Compound** 2** was identified as helioxanthin.

#### 3.1.3. Savinin (**3**)

Light yellow powder, [*α*]_D_^16^ -35.5 (*c* 0.20, CHCl_3_), ESI-MS (*m/z*) 375 [M + Na]^+^; ^1^H-NMR (CDCl_3_, 400 MHz) *δ*: 7.50 (1H, d,* J* = 1.6 Hz, H-7), 7.08 (1H, dd,* J* = 8.0, 1.6 Hz, H-6), 7.03 (1H, d,* J* = 1.6 Hz, H-2), 6.88 (1H, d,* J* = 8.0 Hz, H-5), 6.73 (1H, d,* J* = 8.0 Hz, H-5′), 6.66 (1H, d,* J* = 1.6 Hz, H-2′), 6.64 (1H, dd,* J* = 8.0, 1.6 Hz, H-6′), 6.03 (2H, s, -OCH_2_O-, H-10), 5.93 (2H, m, -OCH_2_O-, H-10′), 4.25 (2H, m, H-9′), 3.75 (1H, m, H-8′), 3.00 (1H, dd,* J* = 14.4, 4.4 Hz, H-7′e), 2.61 (1H, dd,* J* = 14.4, 10.0 Hz, H-7′a); ^13^C-NMR (CDCl_3_, 100 MHz) *δ*: 172.6 (C-9, C=O), 149.3 (C-4), 148.4 (C-3), 148.0 (C-3′), 146.6 (C-4′), 137.4 (C-7), 131.6 (C-1′), 128.3 (C-1), 126.2 (C-6), 125.9 (C-8), 122.2 (C-6′), 109.2 (C-2′), 108.9 (C-5′), 108.8 (C-2), 108.6 (C-5), 101.8 (C-10, -OCH_2_O-), 101.1 (C-10′, -OCH_2_O-), 69.6 (C-9′, lactone, -CH_2_-), 40.0 (C-8′), 37.6 (C-7′). Based on a comparison of these NMR and ESI-MS data with the data reported previously [16], compound** 3** was identified as savinin.

#### 3.1.4. Taiwanin C (**4**)

Light yellow powder, ESI-MS (*m/z*) 371 [M + Na]^+^; ^1^H-NMR (DMSO-*d*_*6*_, 400 MHz) *δ*: 7.95 (1H, s, H-7′), 7.53 (1H, s, H-2′), 7.05 (1H, d,* J* = 8.0 Hz, H-5), 6.90 (1H, s, H-5′), 6.88 (1H, d,* J* = 1.6 Hz, H-2), 6.76 (1H, dd,* J* = 8.0, 1.6 Hz, H-6), 6.19 (2H, d,* J* = 2.0 Hz, H-10′), 6.15, 6.11 (each 1H, d,* J* = 0.8 Hz, H-10), 5.43 (2H, brs, H-9′); ^13^C-NMR (DMSO-*d*_*6*_, 100 MHz) *δ*: 169.8 (C-9, C=O), 150.2 (C-4′), 149.0 (C-3′), 147.6 (C-3), 147.6 (C-4), 140.9 (C-7), 139.3 (C-8′), 134.9 (C-6′), 130.0 (C-1′), 128.9 (C-1), 123.9 (C-6), 119.0 (C-8), 120.2 (C-7′), 111.0 (C-2), 108.6 (C-5), 104.3 (C-2′), 102.7 (C-5′), 102.7 (C-10′, -OCH_2_O-), 101.7 (C-10, -OCH_2_O-), 68.5 (C-9′, lactone, -CH_2_-). All spectral data agreed with those previously reported [17]. Compound** 4** was identified as taiwanin C.

#### 3.1.5. 6-Methoxy-7-Hydroxycoumarin (**5**)

Colorless needles (MeOH), UV 254 nm and 365 nm were blue-fluorescence, ESI-MS (*m/z*) 215 [M + Na]^+^; ^1^H-NMR (CD_3_OD, 400 MHz) *δ*: 7.85 (1H, d,* J* = 9.6 Hz, H-4), 7.09 (1H, s, H-5), 6.76 (1H, s, H-8), 6.20 (1H, d,* J* = 9.6 Hz, H-3), 3.90 (3H, s, -OCH_3_); ^13^C-NMR (CD_3_OD, 100 MHz) *δ*: 162.7 (C-2), 151.7 (C-7), 150.1 (C-9), 145.8 (C-6), 144.8 (C-4), 111.3 (C-3), 111.2 (C-10), 108.7 (C-5), 102.7 (C-8), 55.5 (-OCH_3_). The data of ^1^H- and ^13^C-NMR were consistent with those in previous reports [18, 19]. Compound** 5** was identified as 6*-*methoxy-7-hydroxycoumarin.

#### 3.1.6. Behenic Acid (**6**)

White powder, ESI-MS (*m/z*) 363 [M + Na]^+^; ^1^H-NMR (CDCl_3_, 400 MHz) *δ*: 2.36 (2H, t,* J* = 7.6 Hz, H-2), 1.64 (2H, m, H-3), 1.29-1.25 (36H, brs, 18×CH_2_), 0.88 (3H, t,* J* = 7.2 Hz, CH_3_-22). ^13^C-NMR (CDCl_3_, 100 MHz) *δ*: 179.5 (C-1), 34.0 (C-2), 32.0 (C-3), 29.8-29.1 (C-4~19), 24.8 (C-20), 22.8 (C-21), 14.2 (C-22). The data of ^1^H- and ^13^C-NMR were consistent with those in previous report [19]. Compound** 6** was identified as behenic acid.

#### 3.1.7. 3-O-Caffeoylquinic Acid (**7**)

Yellow amorphous powder; ESI-MS (*m/z*) 377 [M + Na]^+^; ^1^H-NMR (DMSO-*d*_*6*_, 400 MHz) *δ*: 7.48 (1H, d,* J* = 16.0 Hz, H-7′), 7.09 (1H, d,* J* = 1.6 Hz, H-2′), 6.95 (1H, dd,* J* = 8.4, 1.6 Hz, H-6′), 6.79 (1H, d,* J* = 8.0 Hz, H-5′), 6.25 (1H, d,* J* = 16.0 Hz, H-8′), 5.20 (1H, m, H-3), 3.98 (1H, brs, H-5), 3.54 (1H, dd,* J* = 10.0, 2.0 Hz, H-4), 1.88 (2H, m, H-2), 1.73 (1H, br d,* J* = 13.2 Hz, H-6a), 2.06 (1H, br d,* J* = 12.0 Hz, H-6b); ^13^C-NMR (DMSO-*d*_*6*_, 100 MHz) *δ*: 178.2 (C-7), 167.0 (C-9′), 149.5 (C-4′), 146.5 (C-3′), 145.4 (C-7′), 125.9 (C-1′), 121.8 (C-6′), 116.5 (C-5′), 115.4 (C-2′), 114.9 (C-8′), 76.1 (C-1), 73.7 (C-4), 71.9 (C-3), 71.8 (C-5), 40.2 (C-2), 38.6 (C-6). Based on a comparison of these NMR and ESI-MS data with data previously reported, compound** 7** was identified as 3-*O*-caffeoylquinic acid [20–22].

#### 3.1.8. 5-O-Caffeoylquinic Acid (**8**)

Yellow amorphous powder; ESI-MS (*m/z*) 377 [M + Na]^+^; ^1^H-NMR (CD_3_OD, 400 MHz) *δ*: 7.57 (1H, d,* J* = 16.0 Hz, H-7′), 7.05 (1H, d,* J* = 2.0 Hz, H-2′), 6.93 (1H, dd,* J* = 8.4, 1.6 Hz, H-6′), 6.78 (1H, d,* J* = 8.4 Hz, H-5′), 6.29 (1H, d,* J* = 16.0 Hz, H-8′), 5.40 (1H, m, H-5), 4.17 (1H, m, H-3), 3.73 (1H, dd,* J* = 9.6, 3.2 Hz, H-4), 2.18 (2H, m, H-2a, 6a), 2.03 (2H, m, H-2b, 6b); ^13^C-NMR (CD_3_OD, 100 MHz) *δ*: 178.6 (C-7), 167.9 (C-9′), 148.2 (C-4′), 145.7 (C-3′), 145.4 (C-7′), 126.5 (C-1′), 121.7 (C-6′), 115.3 (C-5′), 114.2 (C-2′), 114.0 (C-8′), 76.5 (C-1), 73.3 (C-4), 71.3 (C-3), 71.1 (C-5), 38.9 (C-2), 37.4 (C-6). Based on a comparison of these NMR and ESI-MS data with data previously reported, compound** 8** was identified as 5-*O*-caffeoylquinic acid [18, 20].

#### 3.1.9. 1,3-Di-O-Caffeoylquinic Acid (**9**)

Yellow amorphous powder; ESI-MS (*m/z*) 539 [M + Na]^+^; ^1^H-NMR (DMSO-*d*_*6*_, 400 MHz) *δ*: 7.49 (1H, d,* J* = 15.6 Hz, H-7′′), 7.40 (1H, d,* J* = 15.6 Hz, H-7′), 7.05 (1H, br s, H-2′′), 7.01 (1H, br s, H-2′), 6.94 (1H, d,* J* = 8.0 Hz, H-6′′), 6.90 (1H, d,* J* = 8.0 Hz, H-6′), 6.74 (2H, br s, H-5′′, H-5′), 6.25 (1H, d,* J* = 15.6 Hz, H-8′′), 6.18 (1H, d,* J* = 16.0 Hz, H-8′), 5.28 (1H, br s, H-3), 4.01 (1H, br s, H-5), 3.54 (1H, d,* J* = 8.4 Hz, H-4), 2.58 (1H, m, H-6a), 2.38 (1H, m, H-2a), 2.19 (1H, m, H-6b), 1.81 (1H, m, H-2b); ^13^C-NMR (DMSO-*d*_*6*_, 100 MHz) *δ*: 175.8 (C-7), 166.9 (C-9′′), 165.6 (C-9′), 149.9 (C-4′′), 149.1 (C-4′), 146.6 (C-3′′), 146.5 (C-3′), 145.5 (C-7′′), 144.3 (C-7′), 126.1 (C-1′′), 125.6 (C-1′), 121.9 (C-6′′), 121.2 (C-6′), 116.5 (C-5′′), 116.5 (C-5′), 115.3 (C-2′′), 115.2 (C-2′), 116.5 (C-8′), 114.7 (C-8′′), 82.4 (C-1), 73.2 (C-4), 70.9 (C-3), 69.6 (C-5), 38.3 (C-2), 35.3 (C-6). All spectral data agreed with those previously reported [20, 21]. Compound** 9** was identified as 1,3-di-*O*-caffeoylquinic acid.

#### 3.1.10. 1,4-Di-O-Caffeoylquinic Acid (**10**)

Yellow amorphous powder; ESI-MS (*m/z*) 539 [M + Na]^+^; ^1^H-NMR (DMSO-*d*_*6*_, 400 MHz) *δ*: 7.50 (2H, d,* J* = 15.6 Hz, H-7′, 7′′), 7.02 (2H, m, H-2′, 2′′), 6.95 (2H, m, H-6′, 6′′), 6.75 (2H, m, H-5′, H-5′′), 6.24 (2H, d,* J* = 15.6 Hz, H-8′, 8′′), 5.55 (1H, br s, H-3), 4.98 (1H, br s, H-4), 4.24 (1H, br s, H-5), 2.15 (3H, m, H-6a, 6b, 2a), 1.87 (1H, br s, H-2b); ^3^C-NMR (DMSO-*d*_*6*_, 100 MHz) *δ*: 177.6 (C-7), 166.8 (C-9′′), 166.6 (C-9′), 149.2 (C-4′′), 149.2 (C-4′), 146.2 (C-3′′), 146.2 (C-3′), 146.1 (C-7′′), 146.1 (C-7′), 126.1 (C-1′′), 125.8 (C-1′), 122.0 (C-6′′), 122.0 (C-6′), 116.4 (C-5′′), 116.4 (C-5′), 115.4 (C-2′′), 115.4 (C-2′), 114.2 (C-8′′), 114.2 (C-8′), 76.8 (C-1), 76.1 (C-4), 68.4 (C-3), 69.1 (C-5), 40.2 (C-2), 38.3 (C-6). All spectral data agreed with those previously reported [20, 23]. Compound** 10** was identified as 1,4-di-*O*-caffeoylquinic acid.

#### 3.1.11. 1,5-Di-O-Caffeoylquinic Acid (**11**)

Yellow amorphous powder; ESI-MS (*m/z*) 539 [M + Na]^+^; ^1^H-NMR (DMSO-*d*_*6*_, 400 MHz) *δ*: 7.50 (1H, d,* J* = 16.0 Hz, H-7′′), 7.38 (1H, d,* J* = 16.0 Hz, H-7′), 7.03 (2H, br s, H-2′, 2′′), 6.94 (2H, t,* J* = 8.0 Hz, H-6′, 6′′), 6.73 (2H, t,* J* = 8.0 Hz, H-5′, H-5′′), 6.23 (1H, d,* J* = 16.0 Hz, H-8′′), 6.20 (1H, d,* J* = 16.4 Hz, H-8′), 5.18 (1H, d,* J* = 10.8 Hz, H-5), 3.68 (1H, br s, H-3), 3.65 (1H, br s, H-4), 2.25 (1H, t,* J* = 12.0 Hz, H-6a), 1.98 (3H, m, H-2a, 2b, 6b); ^3^C-NMR (DMSO-*d*_*6*_, 100 MHz) *δ*: 174.2 (C-7), 166.4 (C-9′′), 165.7 (C-9′), 148.9 (C-4′′), 148.6 (C-4′), 146.3 (C-3′′), 146.2 (C-3′), 145.3 (C-7′′), 144.3 (C-7′), 126.3 (C-1′′), 126.1 (C-1′), 121.6 (C-6′′), 121.0 (C-6′), 116.3 (C-5′′), 116.3 (C-5′), 115.5 (C-2′′), 115.4 (C-2′), 115.1 (C-8′′), 116.2 (C-8′), 83.1 (C-1), 71.4 (C-4), 69.3 (C-3), 70.1 (C-5), 40.0 (C-2), 34.0 (C-6). All spectral data agreed with those previously reported [20, 21]. Compound** 11** was identified as 1,5-di-*O*-caffeoylquinic acid.

#### 3.1.12. (+)-Threo-(7R,8R)-Guaiacylglycerol-*β*-Coniferyl Aldehyde Ether (**12**)

White gum; [*α*]_D_^20^ +51.3 (*c* 0.33, MeOH); ESI-MS (*m/z*) 397 [M + Na]^+^; ^1^H-NMR (CD_3_OD, 400 MHz) *δ*: 9.59 (1H, d,* J* = 8.0 Hz, H-9′, -CHO), 7.58 (1H, d,* J *= 15.6 Hz, H-7′), 6.67 (1H, dd,* J *= 15.6, 8.0 Hz, H-8′), 7.17 (1H, dd,* J *= 8.8, 2.0 Hz, H-6′), 6.85 (1H, dd,* J *= 8.0, 2.0 Hz, H-6), 6.99 (1H, d,* J *= 8.8 Hz, H-5′), 6.70 (1H, d,* J *= 8.0 Hz, H-5), 7.22 (1H, d,* J *= 2.0 Hz, H-2′), 7.03 (1H, d,* J *= 2.0 Hz, H-2), 4.55 (1H, m, H-8), 4.82 (1H, d,* J *= 6.0 Hz, H-7), 3.84 (2H, d,* J *= 4.8 Hz, H-9), 3.82 (3H, s, C-3′ -OCH_3_), 3.79 (3H, s, C-3 -OCH_3_); ^13^C-NMR (CD_3_OD, 100 MHz) *δ*: 194.76 (C-9′, -CHO), 154.16 (C-7′), 151.35 (C-4′), 150.38 (C-3′), 147.38 (C-3), 145.79 (C-4), 132.64 (C-1), 127.96 (C-1′), 126.34 (C-8′), 123.06 (C-6′), 119.84 (C-6), 115.87 (C-5′), 114.27 (C-5), 111.36 (C-2′), 110.70 (C-2), 84.09 (C-8), 72.76 (C-7), 61.13 (C-9), 55.27 (C-3′ -OCH_3_), 55.00 (C-3 -OCH_3_). All spectral data agreed with those previously reported [24]. Compound** 12** was identified as (+)-threo-(7*R*,8*R*)-guaiacylglycerol-*β*-coniferyl aldehyde ether.

#### 3.1.13. (+)-Erythro-(7S,8R)-Guaiacylglycerol-*β*-Coniferyl Aldehyde Ether (**13**)

White gum; [*α*]_D_^20^ +148.9 (*c* 0.12, MeOH); ESI-MS (*m/z*) 397 [M + Na]^+^; ^1^H-NMR (CD_3_OD, 400 MHz) *δ*: 9.60 (1H, d,* J* = 8.0 Hz, H-9′, -CHO), 7.61 (1H, d,* J *= 16.0 Hz, H-7′), 6.68 (1H, dd,* J *= 16.0, 8.0 Hz, H-8′), 7.21 (1H, dd,* J *= 8.4, 2.0 Hz, H-6′), 6.86 (1H, dd,* J *= 8.0, 2.0 Hz, H-6), 7.09 (1H, d,* J *= 8.0 Hz, H-5′), 6.75 (1H, d,* J *= 8.4 Hz, H-5), 7.29 (1H, d,* J *= 2.0 Hz, H-2′), 7.03 (1H, d,* J *= 2.0 Hz, H-2), 4.52 (1H, m, H-8), 4.88 (1H, overlapped, H-7), 3.78 (1H, m, H-9a), 3.55 (1H, m, H-9b), 3.91 (3H, s, C-3′ -OCH_3_), 3.81 (3H, s, C-3 -OCH_3_); ^13^C-NMR (CD_3_OD, 100 MHz) *δ*: 194.76 (C-9′, -CHO), 154.11 (C-7′), 151.66 (C-4′), 150.42 (C-3′), 147.56 (C-3), 145.89 (C-4), 132.29 (C-1), 128.07 (C-1′), 126.42 (C-8′), 123.22 (C-6′), 119.32 (C-6), 115.83 (C-5′), 114.51 (C-5), 111.36 (C-2′), 110.37 (C-2), 84.73 (C-8), 72.55 (C-7), 60.75 (C-9), 55.32 (C-3′ -OCH_3_), 55.00 (C-3 -OCH_3_). All spectral data agreed with those previously reported [24]. Compound** 13** was identified as (+)-erythro-(7*S*,8*R*)-guaiacylglycerol-*β*-coniferyl aldehyde ether.

#### 3.1.14. Ferulic Acid (**14**)

Colorless needles (MeOH); ESI-MS (*m/z*) 217 [M + Na]^+^; ^1^H-NMR (CD_3_OD, 400 MHz)* δ*: 7.60 (1H, d,* J* = 15.9 Hz, H-7), 7.17 (1H, d,* J* = 2.0 Hz, H-2), 7.06 (1H, dd,* J* = 8.4, 2.0 Hz, H-6), 6.81 (1H, d,* J* = 8.4 Hz, H-5), 6.32 (1H, d,* J* = 16.0 Hz, H-8), 3.88 (3H, s, -OCH_3_); ^13^C-NMR (CD_3_OD, 100 MHz)* δ*: 169.8 (C-9, -COOH), 149.2 (C-4), 148.0 (C-3), 145.5 (C-7), 126.5 (C-1), 122.6 (C-6), 115.1 (C-8), 114.8 (C-5), 110.4 (C-2), 55.1 (-OCH_3_). All spectral data agreed with those previously reported [25]. Compound** 14** was identified as ferulic acid.

#### 3.1.15. Caffeic Acid (**15**)

Colorless needles (MeOH); ESI-MS (*m/z*) 203 [M + Na]^+^; ^1^H-NMR (DMSO-*d*_*6*_, 400 MHz)* δ*: 7.58 (1H, d,* J* = 16.0 Hz, H-7), 7.17 (1H, d,* J* = 2.0 Hz, H-2), 7.00 (1H, dd,* J* = 8.4, 2.0 Hz, H-6), 6.88 (1H, d,* J* = 8.4 Hz, H-5), 6.33 (1H, d,* J* = 16.0 Hz, H-8); ^13^C-NMR (DMSO-*d*_*6*_, 100 MHz)* δ*: 166.9 (C-9, -COOH), 149.1 (C-4), 146.4 (C-3), 145.3 (C-7), 126.3 (C-1), 121.8 (C-6), 116.4 (C-5), 115.3 (C-8), 115.2 (C-2). All spectral data agreed with those previously reported [26]. Compound** 15** was identified as caffeic acid.

#### 3.1.16. Stigmasterol (**16**)

Colorless needles (MeOH); ^1^H-NMR (CDCl_3_, 400 MHz) *δ*: 5.32 (1H, br s, H-6), 5.15 (1H, dd,* J* = 15.2, 8.4 Hz, H-22), 5.01 (1H, dd,* J* = 15.2, 8.4 Hz, H-23), 3.52 (1H, m, H-3*α*), 0.97 (3H, s, H-19), 0.89 (3H, d,* J* = 6.4 Hz, H-21), 0.82 (3H, d,* J* = 6.4 Hz, H-26), 0.83 (3H, t,* J* = 7.6 Hz, H-29), 0.77 (3H, d,* J* = 6.0 Hz, H-27), 0.65 (3H, s, H-18); ^13^C-NMR (CDCl_3_, 100 MHz) *δ*: 140.9 (C-5), 138.4 (C-22), 129.4 (C-23), 121.8 (C-6), 71.9 (C-3), 57.0 (C-14), 56.1 (C-17), 51.3 (C-24), 50.2 (C-9), 42.4 (C-4), 42.3 (C-13), 40.6 (C-20), 39.8 (C-12), 37.4 (C-1), 36.6 (C-10), 32.0 (C-7), 32.0 (C-8), 32.0 (C-25), 31.7 (C-2), 29.0 (C-16), 25.5 (C-28), 24.4 (C-15), 21.3 (C-11), 21.2 (C-21), 19.5 (C-26), 19.1 (C-19), 19.1 (C-27), 12.3 (C-29), 12.1 (C-18). All spectral data agreed with those previously reported [27]. Compound** 16** was identified as stigmasterol.

#### 3.1.17. *β*-Sitosterol (**17**)

Colorless needles (MeOH); ^1^H-NMR (CDCl_3_, 400 MHz),* δ*: 5.31 (1H, br s, H-6), 3.52 (1H, m, H-3*α*), 0.99 (3H, d,* J* = 6.8 Hz, H-21), 0.97 (3H, s, H-19), 0.82 (3H, d,* J* = 6.4 Hz, H-26), 0.79 (3H, t,* J* = 7.2 Hz, H-29), 0.77 (3H, d,* J* = 6.0 Hz, H-27), 0.66 (3H, s, H-18); ^13^C-NMR (CDCl_3_, 100 MHz) *δ*: 140.9 (C-5), 121.8 (C-6), 71.9 (C-3), 56.9 (C-14), 56.2 (C-17), 50.2 (C-9), 45.9 (C-24), 42.4 (C-4), 42.4 (C-13), 39.9 (C-12), 37.4 (C-1), 36.6 (C-10), 36.2 (C-20), 34.0 (C-22), 32.0 (C-7), 32.0 (C-8), 31.7 (C-2), 29.3 (C-25), 28.3 (C-16), 26.2 (C-23), 24.4 (C-15), 23.2 (C-28), 21.2 (C-11), 19.9 (C-27), 19.5 (C-19), 19.1 (C-21), 18.9 (C-26), 12.1 (C-29), 11.9 (C-18). All spectral data agreed with those previously reported [27]. Compound** 17** was identified as *β*-sitosterol.

#### 3.1.18. Adenosine (**18**)

Colorless sand crystal (MeOH), ESI-MS (*m/z*) 290 [M + Na]^+^; ^1^H-NMR (DMSO-*d*_*6*_, 400 MHz) *δ*: 8.35 (1H, s, H-2), 8.14 (1H, s, H-4), 7.36 (2H, s, -NH_2_), 5.89 (1H, d,* J* = 6.4 Hz, H-1′), 5.46 (1H, d,* J* = 6.0 Hz, 2′-OH), 5.42 (1H, m, 5′-OH), 5.20 (1H, d,* J* = 4.8 Hz, 3′-OH), 4.62 (1H, q,* J* = 6.0 Hz, H-2′), 4.17 (1H, m, H-3′), 3.98 (1H, m, H-4′), 3.69 (1H, m, H-5′a), 3.57 (1H, m, H-5′b); ^13^C-NMR (DMSO-*d*_*6*_, 100 MHz) *δ*: 156.7 (C-1), 152.9 (C-2), 149.6 (C-3), 140.5 (C-4), 119.9 (C-5), 88.5 (C-1′), 86.5 (C-4′), 74.0 (C-2′), 71.2 (C-3′), 62.3 (C-5′). All spectral data agreed with those previously reported [28]. Compound** 18** was identified as adenosine.

#### 3.1.19. Syringin (**19**)

Colorless needles (MeOH), ESI-MS (*m/z*) 395 [M + Na]^+^;^ 1^H-NMR (CD_3_OD, 400 MHz) *δ*: 6.74 (2H, s, H-3, 5), 6.56 (1H, d,* J* = 16.0 Hz, H-7), 6.34 (1H, dt,* J* = 16.0, 5.6 Hz, H-8), 4.87 (1H, overlapped, H-1′), 4.22 (2H, dd,* J* = 5.6, 1.2 Hz, H-9), 3.85 (6H, s, 2OCH_3_), 3.79 (1H, dd,* J* = 12.0, 2.0 Hz, H-6′a), 3.68 (1H, dd,* J* = 12.0, 4.8 Hz, H-6′b), 3.50-3.38 (3H, m, H-3′, 4′, 5′), 3.22 (1H, m, H-2′); ^13^C-NMR (CD_3_OD, 100 MHz) *δ*: 153.0 (C-6), 153.0 (C-2), 134.6 (C-4), 133.9 (C-1), 130.0 (C-7), 128.7 (C-8), 104.1 (C-3), 104.1 (C-5), 104.0 (C-1′), 77.0 (C-5′), 76.5 (C-3′), 74.4 (C-2′), 70.0 (C-4′), 62.3 (C-9), 61.3 (C-6′), 55.7 (2OCH_3_). All spectral data agreed with those previously reported [29, 30]. Compound** 19** was identified as syringin.

#### 3.1.20. Trans-Coniferin (**20**)

White powder, ESI-MS (*m/z*) 365 [M + Na]^+^; ^1^H-NMR (CD_3_OD, 400 MHz) *δ*: 7.10 (1H, d,* J* = 8.4 Hz, H-5), 7.05 (1H, d,* J* = 1.6 Hz, H-2), 6.95 (1H, dd,* J* = 8.4, 1.6 Hz, H-6), 6.55 (1H, d,* J* = 16.0 Hz, H-7), 6.30 (1H, dt,* J* = 16.0, 5.6 Hz, H-8), 4.89 (1H, d,* J* = 7.2 Hz, H-1′), 4.21 (2H, d,* J* = 5.2 Hz, H-9), 3.86 (3H, s, OCH_3_), 3.84 (1H, m, H-6′a), 3.71 (1H, dd,* J* = 12.0, 4.8 Hz, H-6′b), 3.50 (2H, m, H-4′, 5′), 3.41 (2H, m, H-2′, 3′); ^13^C-NMR (CD_3_OD, 100 MHz) *δ*: 149.6 (C-3), 146.3 (C-4), 132.3 (C-1), 130.0 (C-7), 127.6 (C-8), 119.4 (C-6), 116.6 (C-5), 110.0 (C-2), 101.4 (C-1′), 76.9 (C-5′), 76.5 (C-3′), 73.6 (C-2′), 70.0 (C-4′), 62.4 (C-9), 61.2 (C-6′), 55.4 (OCH_3_). All spectral data agreed with those previously reported [30]. Compound** 20** was identified as* trans*-coniferin.

**Figure 1:**
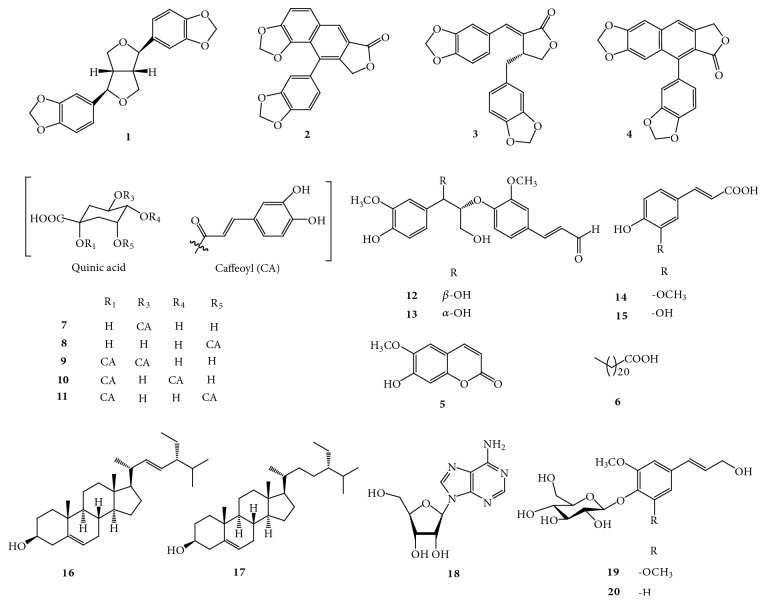
The chemical structures of compounds** 1-20**.

The chemical structures of compounds** 1–20** are shown in Figure 1.

### 3.2. Effects of Compounds on NO and PGE_2_ Production in LPS-Induced BV2 Microglial Cells

Among the isolated compounds from* A. henryi*, we selected 13 compounds (compounds** 1**–**3**,** 5**,** 7**,** 9**,** 10**,** 11**,** 14**,** 15**, and** 18**–**20**) based on their number of publications, and these compounds were screened for anti-inflammatory effects, including the inhibition of NO and PGE_2_ production, in LPS-stimulated BV2 microglial cells. BV2 cells were pretreated with the compounds for 3 h and stimulated with LPS (1 *μ*g/mL) for 24 h. The concentration required to inhibit the production of NO by 50% (IC_50_ value) was calculated based on the concentrations of NO and PGE_2_ released into the culture media as measured by the Griess method and PGE_2_ ELISA kit, respectively. Among the tested compounds, only compound** 3** showed inhibitory effects against LPS-induced NO production with an IC_50_ value of 2.22 ± 0.11 *μ*M and LPS-induced PGE_2_ production with an IC_50_ value of 2.28 ± 0.23 *μ*M. The IC_50_ values of butein, a positive control, were 4.41 ± 0.45 *μ*M in NO production and 3.26 ± 0.53 *μ*M in PGE_2_ production, respectively. At tested concentrations, all test compounds did not show cytotoxic effects to BV2 cells (data not shown). Therefore, compound** 3** was further investigated to elucidate the up-stream signaling pathways of its antineuroinflammatory effect.

### 3.3. Effect of Compound** 3** on LPS-Induced Expression of iNOS and COX-2 Protein in BV2 Microglial Cells

**Figure 2:**
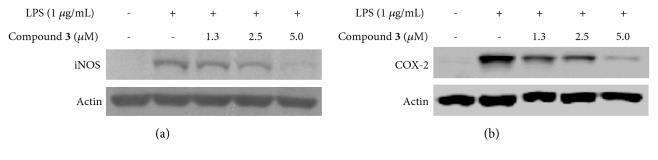
The effect of compound** 3** on LPS-induced iNOS and COX-2 protein expression in BV2 microglial cells. Cells were pretreated with/without the indicated concentrations of compound** 3** for 3 h and then stimulated with LPS (1 *μ*g/mL) for 24 h. The levels of iNOS and COX-2 were determined by Western blot analysis. The experiment was repeated three times, and similar results were obtained.

Inhibition of iNOS and COX-2 protein expression could be an important strategy to prevent neuroinflammation. In the present study, it was investigated whether compound** 3** inhibits LPS-induced overexpression of iNOS and COX-2 protein in BV2 cells. BV2 cells were pretreated with compound** 3** for 3 h and stimulated with LPS (1 *μ*g/mL) for 24 h. As a result, LPS augmented the expression of iNOS and COX-2 proteins; however, pretreatment with compound** 3 **reversed these responses (Figure 2).

### 3.4. Effect of Compound** 3** on LPS-Induced Up-Regulation of Pro-Inflammatory Cytokines

**Figure 3:**
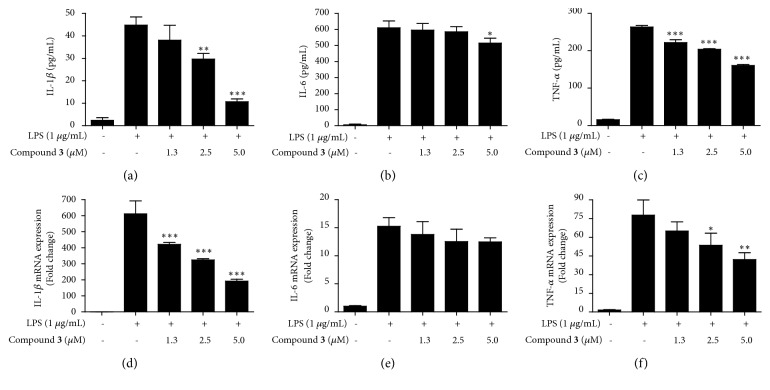
Effects of compound** 3** on LPS-induced production and expression of pro-inflammatory cytokines and their mRNA, IL-1*β* (a, d), IL-6 (b, e), and TNF-*α* (c, f) in BV2 microglial cells. Cells were pretreated with/without the indicated concentrations of compound** 3** for 3 h and then stimulated with LPS (1 *μ*g/mL) for 24 h and 6 h, respectively. The concentrations of cytokines were measured by ELISA, and the mRNA levels of the proinflammatory cytokines were analyzed by qRT-PCR. ^*∗*^*p* < 0.05, ^*∗∗*^*p* < 0.01, and ^*∗∗∗*^*p* < 0.001 in comparison with LPS-treated group.

To investigate whether compound** 3** inhibits the production of LPS-induced proinflammatory cytokines including IL-1*β*, IL-6, and TNF-*α*, BV2 cells were pretreated with compound** 3** for 3 h and stimulated with LPS (1 *μ*g/mL) for 24 h. Pretreatment with compound** 3** attenuated the LPS-induced production of IL-1*β* and TNF-*α*, but it had little effect on IL-6 production (Figures 3(a)–3(c)). Subsequently, to elucidate how compound** 3** inhibits proinflammatory cytokine production, the mRNA levels of the cytokines were examined. BV2 cells were pretreated with compound** 3** for 3 h and stimulated with LPS (1 *μ*g/mL) for 6 h. Consistent with the results obtained from the cytokine production data, pretreatment with compound** 3** reduced the LPS-induced mRNA levels of IL-1*β* and TNF-*α*, but it did not affect to mRNA level of IL-6 (Figures 3(d)–3(f)). These results suggested that compound** 3** exhibits the antineuroinflammatory effects by negatively regulating the production of IL-1*β* and TNF-*α* at the transcriptional level in LPS-challenged BV2 microglial cells.

### 3.5. Effect of Compound** 3** on LPS-Induced Activation of MAPK Pathway in BV2 Cells

**Figure 4:**
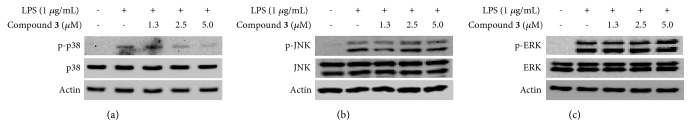
Effects of compound** 3** on the LPS-induced activation of MAPK pathways in BV2 microglial cells. Lysates were prepared from cells pretreated with/without the indicated concentrations of compound** 3** for 3 h and then with LPS (1 *μ*g/mL) for 30 min. The levels of p-p38, p38, p-ERK, ERK, p-JNK, and JNK were determined by Western blot analysis. Actin was used as a loading control. The experiment was repeated three times, and similar results were obtained.

We examined whether the antineuroinflammatory effects of compound** 3** contribute to the inactivation of MAPK in the present study. BV2 cells were pretreated with compound** 3** for 3 h and stimulated with LPS (1 *μ*g/mL) for 30 min. LPS markedly increased the phosphorylation of p38, JNK, and ERK. Pretreatment with compound** 3** inhibited the phosphorylation of p38 (Figure 4(a)), but it had no effect on the phosphorylation of JNK and ERK (Figures 4(b) and 4(c)).

## 4. Discussion

The present study demonstrated that compound** 3** (savinin), one of the isolated compounds from the roots of* A. henryi* exerted antineuroinflammatory effects in LPS-treated microglial cells. Compound** 3** inhibited LPS-induced the release of proinflammatory mediators, including NO, PGE_2_, iNOS, COX-2, IL-1*β*, and TNF-*α*. These inhibitory effects of compound** 3** were mediated by the inactivation of the p38 MAPK pathway.

NO and PGE_2_, as well as their regulatory enzymes iNOS and COX-2, are important mediators for neuroinflammation. They are involved in the pathogenesis of various neuroinflammatory pathophysiological conditions [31]. In addition, proinflammatory cytokines, including IL-1*β*, IL-6, and TNF-*α*, are important factors in CNS immune responses, and mediate neurodegeneration [3]. Microglial cells are activated by various stimuli. The activation of these cells leads to the release of these proinflammatory mediators and the exacerbation of neuroinflammation and neuroglia-induced neurotoxicity [31, 32]. Our investigation showed that compound** 3, **isolated from* A. henryi*, suppressed NO and PGE_2_ production in LPS-stimulated BV2 microglial cells and also inhibited the LPS-induced expression of the iNOS and COX-2 protein (Figure 2). Additionally, the present data indicated that compound** 3** significantly suppressed the release of IL-1*β* and TNF-*α*, but not IL-6, at the protein and mRNA levels (Figure 3).

MAPKs are a family of serine/threonine protein kinases, and they consist of three major subunits: p38, extracellular signal-regulated kinase (ERK), and c-Jun N-terminal kinase (JNK). MAPKs are involved in various cellular processes, including proliferation, differentiation, stress responses, and immune responses [33]. In inflammatory responses, the activation of MAPKs leads to release of inflammatory-related factors, such as iNOS, COX-2, ILs, and TNF-*α* [34]. Therefore, we further investigated whether the antineuroinflammatory effects of compound** 3** in LPS-stimulated BV2 microglial cells were related to the inactivation of MAPKs. Our results showed that compound** 3** significantly inhibited the phosphorylation of p38 MAPK, but it did not affect the phosphorylation of ERK and JNK MAPKs.

Nuclear factor kappa B (NF-*κ*B) is one of the major transcription factors regulating inflammatory responses. When microglial cells are stimulated with ILs, TNF-*α*, interferons, or LPS, NF-*κ*B can be activated following increased translocation of p65 and p50, which are subunits of the NF-*κ*B dimer, into the nucleus, as well as the phosphorylation and degradation of inhibitor of kappa B (I*κ*B)-*α* in the cytosol [35]. This response upregulates the expression of proinflammatory cytokines, chemokines, and adhesion molecules in microglial cells [1]. Therefore, NF-*κ*B can be an important target for the treatment of neuroinflammation-related neurodegenerative diseases. Interestingly, pretreatment with compound** 3** did not decrease the nuclear translocation of p65 and p50 or the phosphorylation and degradation of I*κ*B-*α* (data not shown). Considering the effect of compound** 3** on the LPS-induced activation of NF-*κ*B and MAPK pathways, it is suggested that compound** 3** exerted antineuroinflammatory effects by specifically inactivating the p38 MAPK pathway.

Various pharmacological activities of lignans, including compound** 3** (savinin), have been reported. These activities include cytotoxic effects against human cancer cell lines [36], CYP3A4 inactivation [37], antisevere acute respiratory syndrome associated coronavirus (SARS-CoV) activity [38], inhibition of PGE_2_ production in rat peritoneal macrophages [39], antiestrogenic [40], insecticidal [41], and inhibition of TNF-*α* production in RAW264.7 macrophage cells [42]; however, this is the first finding on the antineuroinflammatory effects of compound** 3** (savinin) to the best of our knowledge.

## 5. Conclusions

In summary, 20 secondary metabolites were isolated from the EtOAc-, butanol-, and PE-soluble fractions of the methanol extract of* Acanthopanax henryi* roots. Among these compounds, savinin (compound** 3**) was the only compound which inhibited the production of NO and PGE_2_, and the expression of iNOS and COX-2 proteins in LPS-stimulated BV2 microglial cells. In addition, this compound significantly suppressed the release of IL-1*β* and TNF-*α* at the protein and mRNA levels. These inhibitory effects of compound** 3** were mediated by p38 MAPK, but not ERK and JNK MAPKs. Taken together, our investigation showed that compound** 3** can be a promising candidate for the development of treatment options for neurodegenerative diseases. Further studies elucidating the detailed mechanisms underlying the anti-inflammatory effects of compound** 3** are suggested.

## Data Availability

The data used to support the findings of this study are available from the corresponding author upon request.

## References

[1] Yuan L., Liu S., Bai X. (2016). Oxytocin inhibits lipopolysaccharide-induced inflammation in microglial cells and attenuates microglial activation in lipopolysaccharide-treated mice. *Journal of Neuroinflammation*.

[2] Zhang F., Wang Y.-Y., Yang J., Lu Y.-F., Liu J., Shi J.-S. (2013). Tetrahydroxystilbene glucoside attenuates neuroinflammation through the inhibition of microglia activation. *Oxidative Medicine and Cellular Longevity*.

[3] Kempuraj D., Thangacel R., Natteru P. A. (2016). Neuroinflammation induces neurodegeneration. *Journal of Neurology, Neurosurgery and Spine*.

[4] Li B., Lee D.-S., Jeong G.-S., Kim Y.-C. (2012). Involvement of heme oxygenase-1 induction in the cytoprotective and immunomodulatory activities of 6,4′-dihydroxy-7-methoxyflavanone in murine hippocampal and microglia cells. *European Journal of Pharmacology*.

[5] Kim J.-H., Liu X.-Q., Dai L., Yook C.-S., Lee K.-T. (2014). Cytotoxicity and anti-inflammatory effects of root bark extracts of Acanthopanax henryi. *Chinese Journal of Natural Medicines*.

[6] Zhang X. D., Liu X. Q., Kim Y. H., Whang W. K. (2014). Chemical constituents and their acetyl cholinesterase inhibitory and antioxidant activities from leaves of Acanthopanax henryi: potential complementary source against Alzheimer's disease. *Archives of Pharmacal Research*.

[7] Hu X., Zhang W., Zhu Q. (1999). *Editorial board of the State Administration of Traditional Chinese Medicine, "Chinese Materia Medica (Zhong Hua Ben Cao)"*.

[8] Zhang X.-D., Li Z., Liu G.-Z. (2016). Quantitative determination of 15 bioactive triterpenoid saponins in different parts of Acanthopanax henryi by HPLC with charged aerosol detection and confirmation by LC-ESI-TOF-MS. *Journal of Separation Science*.

[9] Li Z., Li X. J., Kwon O. K. (2015). Chemical constituents from leaves of Acanthopanax henryi (II). *Natural Product Sciences*.

[10] Liu H. Y., Kim J. H., Liu X. Q., Yook C. S. (2012). Study on chemical constituents from root barks of Acanthopanax henryi. *Journal of Traditional Chinese Medicine University of Hunan*.

[11] Kang D.-H., Kang O.-H., Li Z. (2016). Anti-inflammatory effects of Ciwujianoside C3, extracted from the leaves of Acanthopanax henryi (Oliv.) Harms, on LPS-stimulated RAW 264.7 cells. *Molecular Medicine Reports*.

[12] Han Y.-H., Li Z., Um J.-Y., Liu X. Q., Hong S.-H. (2016). Anti-adipogenic effect of Glycoside St-E2 and Glycoside St-C1 isolated from the leaves of Acanthopanax henryi (Oliv.) Harms in 3T3-L1 cells. *Bioscience, Biotechnology, and Biochemistry*.

[13] Quang T. H., Ngan N. T. T., Yoon C.-S. (2015). Protein tyrosine phosphatase 1b inhibitors from the roots of cudrania tricuspidata. *Molecules*.

[14] Ko W., Sohn J. H., Jang J.-H. (2016). Inhibitory effects of alternaramide on inflammatory mediator expression through TLR4-MyD88-mediated inhibition of NF-кB and MAPK pathway signaling in lipopolysaccharide-stimulated RAW264.7 and BV2 cells. *Chemico-Biological Interactions*.

[15] Kim K.-W., Quang T. H., Ko W. (2018). Anti-neuroinflammatory effects of cudraflavanone a isolated from the chloroform fraction of Cudrania Tricuspidata root bark. *Pharmaceutical Biology*.

[16] Yu K., Song Y., Lu Y., Xiong Z. L., Li F. M. (2012). Phenylpropanoids from Roots of Acanthopanax sessiliflorus (Rupr. et Maxim.) Seem. *Natural Product Research and Development*.

[17] Kocsis L. S., Brummond K. M. (2014). Intramolecular dehydro-diels-alder reaction affords selective entry to arylnaphthalene or aryldihydronaphthalene lignans. *Organic Letters*.

[18] Lin L. C., Yang L. L., Chou C. J. (2002). Constituents from the stems of Ecdysanthera rosea. *Journal of Chinese Medicine*.

[19] Chi J., Li B. C., Zhang M. (2015). Chemical Constituents from Artemisia austro - yunnanensis. *Journal of Kunming University of Science and Technology (Natural Science Edition)*.

[20] Ma Y.-C., Wang X.-Q., Hou F. (2011). Rapid resolution liquid chromatography (RRLC) analysis and studies on the stability of Shuang-Huang-Lian preparations. *Journal of Pharmaceutical and Biomedical Analysis*.

[21] Li J., Yu D. (2011). Chemical constituents from herbs of Erigeron breviscapus. *Zhongguo Zhongyao Zazhi*.

[22] Kwon H. C., Jung C. M., Shin C. G. (2000). A new caffeoyl quinic acid from Aster scaber and its inhibitory activity against human immunodeficiency virus-1 (HIV-1) integrase. *Chemical & Pharmaceutical Bulletin*.

[23] Kim W. K., Bach D.-H., Ryu H. W. (2017). Cytotoxic activities of Telectadium dongnaiense and its constituents by inhibition of the Wnt/*β*-catenin signaling pathway. *Phytomedicine*.

[24] Huang X.-X., Zhou C.-C., Li L.-Z. (2013). The cytotoxicity of 8-O-4′ neolignans from the seeds of Crataegus pinnatifida. *Bioorganic & Medicinal Chemistry Letters*.

[25] Luo X., Wang X.-J., Zhao Y.-W. (2016). Chemical constituents from Notopterygium incisum. *Chinese Traditional and Herbal Drugs*.

[26] Huo L.-N., Wang W., Liu Y. (2016). Chemical constituents from leaves of Perilla frutescens. *Chinese Traditional and Herbal Drugs*.

[27] Ogunlaja O. O., Moodley R., Baijnath H., Jonnalagadda S. B. (2016). Chemical constituents and in vitro antioxidant activity of crude extracts and compounds from leaves and stem bark of Ficus burttdavyi. *Acta Poloniae Pharmaceutica*.

[28] Pan Y. L., Chen H., Li J., Li X., Chen J. W. (2014). Chemical constituents from extract of Isatidis Radix. *Zhongchengyao*.

[29] Niwa M., Iwadare Y., Wu Y.-C., Hirata Y. (1988). Two new phenylpropanoid glycosides from wikstroemia sikokiana. *Chemical & Pharmaceutical Bulletin*.

[30] Sugiyama M., Nagayama E., Kikuchi M. (1993). Lignan and phenylpropanoid glycosides from Osmanthus asiaticus. *Phytochemistry*.

[31] Guo C., Yang L., Wan C.-X. (2016). Anti-neuroinflammatory effect of Sophoraflavanone G from Sophora alopecuroides in LPS-activated BV2 microglia by MAPK, JAK/STAT and Nrf2/HO-1 signaling pathways. *Phytomedicine*.

[32] Vane J. R., Mitchell J. A., Appleton I. (1994). Inducible isoforms of cyclooxygenase and nitric-oxide synthase in inflammation. *Proceedings of the National Acadamy of Sciences of the United States of America*.

[33] Liu Y., Shepherd E. G., Nelin L. D. (2007). MAPK phosphatases-regulating the immune response. *Nature Reviews Immunology*.

[34] Velagapudi R., Aderogba M., Olajide O. A. (2014). Tiliroside, a dietary glycosidic flavonoid, inhibits TRAF-6/NF-*κ*B/p38-mediated neuroinflammation in activated BV2 microglia. *Biochimica et Biophysica Acta (BBA) - General Subjects*.

[35] Im E. J., Kim S. J., Hong S. B., Park J.-K., Rhee M. H. (2016). Anti-inflammatory activity of bee venom in bv2 microglial cells: mediation of MyD88-dependent NF-*κ*B signaling pathway. *Evidence-Based Complementary and Alternative Medicine*.

[36] Woo K. W., Choi S. U., Park J. C., Lee K. R. (2011). A new lignan glycoside from Juniperus rigida. *Archives of Pharmacal Research*.

[37] Yoo H. H., Lee S.-H., Jin C., Kim D.-H. (2008). Mechanism-based inactivation of cytochrome P450 3A4 by methylenedioxyphenyl lignans from Acanthopanax chiisanensis. *Planta Medica*.

[38] Wen C.-C., Kuo Y.-H., Jan J.-T. (2007). Specific plant terpenoids and lignoids possess potent antiviral activities against severe acute respiratory syndrome coronavirus. *Journal of Medicinal Chemistry*.

[39] Lee S., Ban H. S., Kim Y. P. (2005). Lignans from Acanthopanax chiisanensis having an inhibitory activity on prostaglandin E2 production. *Phytotherapy Research*.

[40] Lee S., Hye H. Y., Xiang L. P., Ju S. K., Sam S. K., Kuk H. S. (2005). Anti-estrogenic activity of lignans from Acanthopanax chiisanensis root. *Archives of Pharmacal Research*.

[41] Nissanka A. P. K., Karunaratne V., Bandara B. M. R. (2001). Antimicrobial alkaloids from Zanthoxylum tetraspermum and caudatum. *Phytochemistry*.

[42] Cho J. Y., Park J., Kim P. S., Yoo E. S., Baik K. U., Park M. H. (2001). Savinin, a lignan from Pterocarpus santalinus inhibits tumor necrosis factor-*α* production and T cell proliferation. *Biological & Pharmaceutical Bulletin*.

